# Making weight makes sense: relative performance gains after rapid weight loss in powerlifting: a randomized controlled trial

**DOI:** 10.1080/15502783.2025.2550309

**Published:** 2025-08-27

**Authors:** Arkadiusz Matras, Michał Czapla, Artur Struzik

**Affiliations:** a Dietetyka #NieNaŻarty, Dietary Counseling Center, Wrocław, Poland; b Wroclaw Medical University, Division of Scientific Research and Innovation in Emergency Medical Service, Department of Emergency Medical Service, Faculty of Nursing and Midwifery, Wroclaw, Poland; c University of La Rioja, Group of Research in Care (GRUPAC), Faculty of Health Sciences, Logroño, Spain; d Wroclaw University of Health and Sport Sciences, Department of Physiology and Biomechanics, Wroclaw, Poland

**Keywords:** Rehydration, strength performance, weight-class sports, IPF score, muscular recovery

## Abstract

**Background:**

Rapid weight loss (RWL) is a common strategy among competitive powerlifters aiming to qualify for lower weight categories and improve competitive advantage. However, the effects of RWL followed by short-term recovery on maximal strength performance remain unclear. This study aimed to examine whether a ~ 5% RWL protocol followed by a 2-hour recovery period affects changes in maximal and relative strength performance in trained male powerlifters.

**Methods:**

This randomized controlled trial (RCT) was registered in the Australian New Zealand Clinical Trials Registry (ACTRN12622000924752). In this RCT study, 26 male powerlifters (24.6 ± 4.5 y; 92.8 ± 13.6 kg) were assigned to a rapid weight loss (RWL; *n* = 13) or control group (CON; *n* = 13). RWL participants followed a 4-day protocol including caloric restriction (−10%), low carbohydrate intake ( <50 g/day), low sodium, and fluid manipulation, targeting a 5% body mass reduction. The CON group maintained habitual diet and hydration. Maximal strength was assessed via simulated powerlifting competition conducted before (C1) and after (C2) the intervention. Performance variables included squat (SQ), bench press (BP), deadlift (DL), and total load (TOTAL), along with IPF GL (International Powerlifting Federation GoodLift points) and IPF GL BP (International Powerlifting Federation GoodLift for Bench Press) scores. Body composition and rate of perceived exertion (RPE) were also evaluated.

**Results:**

The RWL group achieved a mean body mass reduction of 4.81%, with significant decreases in fat mass (−15.7%), fat-free mass (−2.36%), and body water (−2.41%) compared to CON (all *p* < 0.01). Despite these changes, no significant differences in maximal strength (SQ, BP, DL, TOTAL) were observed between C1 and C2 or between groups. However, the RWL group showed a significant post-intervention increase in IPF GL (*p* = 0.015) and IPF GL BP scores (*p* = 0.017). RPE values showed no consistent or practically relevant changes.

**Conclusion:**

In the group that underwent a rapid weight loss of approximately 5%, followed by a short-term recovery period, maximal strength performance was maintained. This indicates that it is possible to reduce body mass without compromising absolute strength levels in competitive powerlifters. At the same time, RWL group achieved higher IPF GL and IPF GL BP values.

## 1. Introduction

Powerlifting consists of three lifts: squat (SQ), bench press (BP), and deadlift (DL). During competition, each athlete has up to three attempts per lift, and the total score (TOTAL) is calculated as the sum of the highest successful attempts in each lift, according to the rules of the organizing federation [1]. As a weight-class sport, powerlifting allows athletes to engage in rapid weight loss (RWL) to qualify for a lower weight category. For example, an athlete typically weighing 96 kg may reduce their body mass by ~3 kg within 3–7 days to compete in the ≤93 kg category instead of the ≤105 kg class [2]

There is a strong positive correlation between muscle mass and powerlifting performance [3,4]. Competing in a lower weight class may offer strategic advantages by placing the athlete among competitors with potentially lower TOTALs. RWL, typically performed over 3–7 days, differs from fat loss, as it primarily targets reductions in gut content, muscle glycogen, and body water rather than adipose tissue [5]. Each of these components may affect performance differently. Reductions in gut content and glycogen stores appear to have minimal impact on maximal strength [6], whereas significant dehydration may impair muscle strength [7,8]. To date, limited evidence exists regarding the combined impact of multiple RWL strategies on powerlifting performance. Most research has examined the effects of single methods, such as ketogenic diets or passive dehydration, on strength outcomes [6,8].

Nonetheless, RWL is widely practiced among powerlifters [2,9]. In the International Powerlifting Federation (IPF), athletes weigh in two hours prior to competition, allowing for short-term recovery using targeted rehydration and energy replenishment strategies. Properly implemented recovery protocols may reverse the potential negative effects of RWL [10].

The aim of this study was to examine whether a ~ 5% rapid weight loss protocol followed by a short-term (2-hour) recovery period affects changes in maximal strength and relative performance (IPF GL scores) in trained male powerlifters. A 5% body mass reduction is considered the upper limit of acceptability by the Australian Institute of Sport, particularly in contexts where the recovery time between weigh-in and competition is less than 4 hours [11]. In scientific literature, a 5% RWL is the most commonly reported value and serves as a reference point in research focused on optimizing body composition in athletes [5]. Mauricio et al. [12] reported that in combat sports the body mass loss up to 5% have impact on competitive performance, confirming its relevance as a standardized threshold.

## 2. Metodology

### 2.1. Participants

Thirty male powerlifters from Poland (Central Europe) completed were recruited via online announcements on powerlifting-related platforms. Twenty-six participants completed the study (age: 24.6 ± 4.5 years; body mass: 92.8 ± 13.6 kg), while four were excluded due to illness. The following inclusion criteria were applied: male sex, a minimum of two years of powerlifting experience, and at least one participation in a powerlifting competition. Competitive experience was defined as participation in at least one official powerlifting competition at the national level. Exclusion criteria included the use of performance-enhancing substances listed by the World Anti-Doping Agency (WADA) and any medical contraindications. The use of such substances was self-reported by the participants. The athletes were accustomed to RWL. Prior to enrollment in the study, their self-reported maximum RWL before competition averaged approximately 4 kg, corresponding to 4.57 ± 1.42% of body mass (median: 4.24%). Participants were randomly assigned (1:1 ratio) to an experimental group (RWL) or control group (CON). The RWL group underwent a rapid weight loss protocol (~5% body mass), while the CON group maintained their habitual dietary practices.

### 2.2. Study design

This was an open-label, randomized, controlled, parallel-group trial registered in the Australian New Zealand Clinical Trials Registry (ACTRN12622000924752). During the first week, all participants reduced their training volume by 50% and performed two training sessions. On days 4–6, they maintained a dietary log. Participants were instructed to follow their habitual normocaloric diet that would not induce significant changes in body mass (days 1–9). On day 7, anthropometric measurements were taken at 9:00, followed by the completion of the UMACL mood questionnaire, and then the first simulated powerlifting meet (C1) at 11:00, during which maximal strength was assessed. Assessors were not blinded to group allocation; however, all performance tests were evaluated in accordance with the judging criteria outlined by the International Powerlifting Federation (IPF) to minimize potential bias. Participants continued their habitual diet on days 8–9.

Between days 10–13, the RWL group implemented a 5% body mass reduction protocol based on the Cunningham method [13], with an energy deficit of 10%, dietary fiber intake below 10 g/day, carbohydrates below 50 g/day, and sodium below 1000 mg/day. Fluid intake was set at 100 ml/kg of body mass from days 10–12 and 15 ml/kg on day 13, following [14] If body mass exceeded the target threshold by more than 0.7% on day 13, dry sauna sessions were employed in 15-minute intervals. The CON group maintained their normal diet and completed a dietary log during this period.

**Figure 1.**
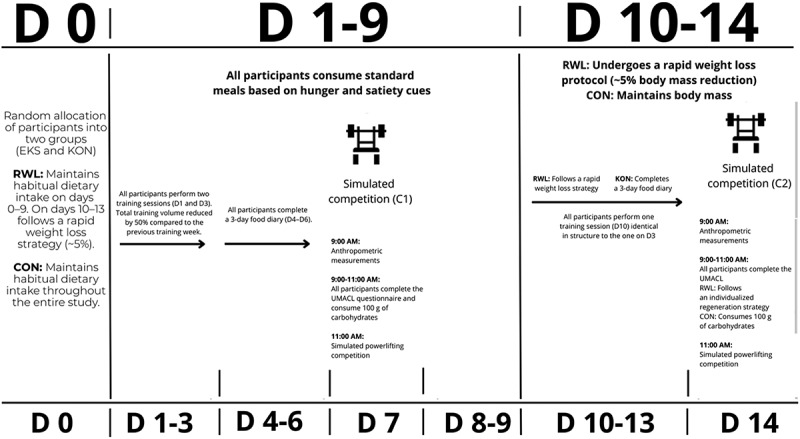
Study protocol.

On day 14, anthropometric measurements were repeated. Between these measurements and the simulated meet, participants completed the UMACL mood questionnaire. The RWL group followed a standardized recovery protocol including oral rehydration solutions (ORS) and carbohydrate-based snacks. At 11:00, a second simulated meet (C2) was held, during which food and fluid intake was allowed ad libitum (Figure 1).

#### 2.2.1. Maximal strength testing

Maximal strength was assessed using simulated powerlifting meets, modeled after the competitions format described by Durguerian et al. [15] During both C1 and C2, participants performed three attempts in the squat (SQ), bench press (BP), and deadlift (DL). The TOTAL was calculated as the sum of the heaviest successful attempt in each lift, according to IPF rules.

#### 2.2.2. Dietary assessment

Dietary intake was evaluated using 3-day food records, analyzing total energy, macronutrient intake, fiber, and sodium content. Data were analyzed using the USDA FoodData Central database [16]. Food intake was tracked via the Fitatu app (Fitatu sp. z o.o., Poznań, Poland).

#### 2.2.3. Measurements

Measurements were taken on days 7 and 14. Body composition was assessed via bioelectrical impedance analysis (BIA) using the InBody570 device (Seoul, Korea), which utilizes a multi-frequency (5, 50, and 500 kHz) current of 400 μA and an 8-point tactile electrode system (feet and hands). Participants were assessed in the morning, in a fasted state, and after voiding, which is consistent with standardized hydration protocols for BIA-based assessments. Participants wore only underwear (boxer briefs) during the assessment. The test was performed in accordance with the manufacturer’s instructions, with legs apart and arms fully extended and abducted from the torso. Each measurement lasted approximately 50 s [17].

#### 2.2.4. Rate of perceived exertion (RPE)

The 10-point Rate of Perceived Exertion (RPE) scale based on Repetitions in Reserve (RIR) was used to assess subjective effort. A score of RPE = 10 was recorded if the athlete could not perform any additional repetitions; RPE = 9 for one more possible repetition; and RPE = 8 for two more possible repetitions [18].

#### 2.2.5. Mood

The Polish version of the University of Wales Institute of Science and Technology Mood Adjective Checklist (UMACL) was used to measure the mood of participants [19,20]. UMALC consists of 29 adjectives divided into three subscales: Tense Arousal (TA), Energetic Arousal (EA), and Hedonic Tone (HT). Each adjective is rated on a four-point scale, with higher scores indicating greater levels of tension, energy, or positive mood, respectively. The Polish version of the tool was carried out by Goryńska. Cronbach’s alpha coefficients for the UMACL subscales were: 0.83 for TA, 0.75 for EA, and 0.88 for HT [19].

### 2.3. Ethics approval and consent to participate

The study was conducted in accordance with the Declaration of Helsinki and was approved by the Senate’s Research Bioethics Commission of the Wroclaw University of Health and Sport Sciences, Poland (approval no. 18/2021). All participants were informed about the aims, procedures, and potential risks of the study. Each individual provided written informed consent prior to participation and was informed of their right to withdraw from the study at any time without providing a reason.

### 2.4. Statistical analysis

The required sample size was calculated using Statistics 13 software based on data from Durguerian et al. [15], indicating a need for 13 subjects per group with an assumed effect size, alpha level of 0.05, and 80% statistical power. To allow for a potential dropout rate of 15%, 30 participants were recruited, of which 26 completed the study. Randomization was performed using a generator from random.org. The distribution of data were checked using the Shapiro – Wilk (W) test. Due to the lack of close-to-normal distributions, non-parametric tests were used. Categorical variables were compared using the chi-square or Fisher’s exact test. Continuous variables were analyzed using the Mann – Whitney U test. Relationships between variables were evaluated using Spearman’s rank correlation. Within-group differences (pre – post) were assessed using the Wilcoxon signed-rank test.

To enhance the interpretability of both within- and between-group differences, effect size estimates were calculated. For within-group comparisons (e.g. C1 vs. C2), we used the matched-pairs rank biserial correlation coefficient (r_c), appropriate for nonparametric paired-sample data. For between-group comparisons, Cliff’s delta (δ) was calculated. Both indices range from − 1 to 1 and are interpreted similarly to correlation coefficients, where values closer to ± 1 indicate stronger effects. According to commonly used thresholds, Cliff’s delta values above 0.43 were considered large, below 0.28 small, and intermediate values moderate. For r_c, values above 0.5 were treated as strong, and below 0.3 as weak. Due to the small sample size, all effect sizes should be interpreted with caution. Full results are available in Supplementary Table S1-S5. Statistical analysis was conducted using R software (version 4.3.0).

## 3. Results

### 3.1. Characteristics of the population sample

The RWL and CON groups were homogeneous and did not significantly differ in body mass, fat mass, fat-free mass (FFM), or body fat percentage at baseline (Table 1).

**Table 1.** Baseline characteristics of participants in the rapid weight loss (RWL) and control (CON) groups.

Variable	Group	Mean	SD	*p*
Age (years)	RWL	26	5.2	0.141
	CON	23.2	3.4	
Height [m]	RWL	1.8	0.07	0.719
	CON	1.8	0.08	
Body Mass [kg]	RWL	94.3	14.2	0.762
	CON	91.3	13.4	
TOTAL [kg]	RWL	567	84.3	0.293
	CON	537	61.1	
Body fat percentage [%]	RWL	19.5	6.21	0.33
	CON	17	5.85	
Training experience [years]	RWL	3.7	1.4	0.714
	CON	3.5	1.6	
Competition experience (number of competitions)	RWL	2.9	1.8	0.579
	CON	2.9	2.4	

Abbreviations: SD – standard deviation; *p* – probability level for differences between RWL and CON groups; RWL – rapid weight loss group; CON – control group.

### 3.2. Rapid weight loss and body composition

The experimental group (RWL) showed significantly greater changes in body composition compared to the control group (CON), including reductions in body mass (−4.81%), total body water (−2.41%), fat mass (−15.7%), fat-free mass (−2.36%), and body fat percentage (−11.5%). In contrast, the CON group exhibited no statistically significant changes in these variables (Table 2).

**Table 2.** Effects of rapid weight loss on body composition variables.

Variable	Group	Timepoint	Mean	SD	Δ±SD	Median	Min	Max	*p*
Body Mass [kg]	RWL	C1	94.3	14.2	−4.81% ± 1.99	92.0	75.1	121	0.002 *
		C2	89.9	14.6		85.5	70.3	116	
	CON	C1	91.3	13.4	−0.17% ± 1.04	93.3	69.8	117	0.784
		C2	91.2	13.7		92.9	68.6	118	
					*p* < 0.001*				
Total body water [%]	RWL	C1	55.1	7.44	−2.41% ± 1.98	54.1	42.1	66.8	0.003*
		C2	53.8	7.49		52.2	41.3	66.1	
	CON	C1	55.1	7.32	0.76% ± 1.42	58.6	42.6	67.1	0.099
		C2	55.5	7.72		57.9	42.4	68.8	
					*p* < 0.001*				
Fat mass [kg]	RWL	C1	18.8	8.06	−15.7% ± 6.75	18.3	9.7	40.5	0.002*
		C2	16.1	7.83		15.4	7.7	36.3	
	CON	C1	15.8	6.41	−4.85% ± 5.01	16.9	7.3	26.8	0.004*
		C2	15.1	6.33		14.4	7.00	26.2	
					*p* < 0.001*				
Fat-free mass [kg]	RWL	C1	75.5	10.3	−2.36% ± 1.76	74.6	57.8	91.9	0.003*
		C2	73.7	10.3		72.2	56.8	90.9	
	CON	C1	75.5	10.1	0.75% ± 1.36	80.4	58.2	92.4	0.1
		C2	76.1	10.6		79.6	58.0	94.6	
					*p* < 0.001*				
Body fat [%]	RWL	C1	19.5	6.21	−11.5% ± 5.45	18.3	11.5	33.5	0.002*
		C2	17.5	6.27		17.0	9.7	31.2	
	CON	C1	17.0	5.85	−4.58% ± 4.65	17.5	9.5	26.6	0.002*
		C2	16.3	5.85		15.3	9.3	26.4	
					*p* < 0.001*				

Abbreviations: SD – standard deviation; Min – minimum value; Max – maximum value; *p* – probability level for differences between RWL and CON groups; RWL – rapid weight loss group; CON – control group; FFM – fat-free mass; Δ±SD – difference between competition 1 (C1) and competition 2 (C2).; * – statistically significant difference (*p* < 0.05).

### 3.3. Rapid weight loss and maximal strength performance

There were no statistically significant differences in maximal strength performance (SQ, BP, DL, TOTAL) between the first (C1) and second (C2) simulated competitions or between the RWL and CON groups. However, the RWL group demonstrated significantly higher IPF GL and IPF GL BP scores in C2 (*p* < 0.05), indicating an advantage due to reduced body mass (Table 3).

**Table 3.** Effects of rapid weight loss on maximal strength performance.

Variable	Group	Timepoint	Mean	SD	Δ±SD	Median	Min	Max	*p*
SQ [kg]	RWL	C1	190	30.9	−0.7% ± 2.13	185	135	260	0.221
		C2	189	28.1		185	140	250	
	CON	C1	192	21.6	1.26% ± 4.13	190	158	215	0.382
		C2	194	22.3		192	155	225	
					*p* = 0.191				
BP [kg]	RWL	C1	125	25.7	0.18% ± 3.1	118	80	170	1
		C2	125	26.7		120	80	180	
	CON	C1	122	15.3	0.48% ± 2.42	120	102	160	0.469
		C2	122	16.1		122	100	160	
					*p* = 0.792				
DL [kg]	RWL	C1	224	34.5	−0.12% ± 4.03	215	180	292	0.822
		C2	224	35.3		210	182	295	
	CON	C1	220	26.0	0.14% ± 3.49	215	178	265	1
		C2	220	23.9		218	180	265	
					*p* = 0.679				
TOTAL [kg]	RWL	C1	540	82.3		530	395	682	0.533
		C2	538	82.5	−0.29% ± 2.19	525	410	700	
	CON	C1	534	55.1	0.58% ± 1.84	530	438	625	0.282
		C2	537	54.2		532	440	625	
					*p* = 0.099				
IPF GL [pkt]	RWL	C1	70.5	9.9	3.83% ± 6.31	68.5	55.1	90.0	0.011*
		C2	73.1	10.4		72.4	54.2	92.4	
	CON	C1	71.3	7.05	−1.23% ± 5.57	71.9	58.8	84.3	0.969
		C2	70.3	7.28		72.9	57.2	81.2	
					*p* = 0.015*				
IPF GL BP [pkt]	RWL	C1	59.3	11.7	4.37% ± 6.79	58.2	42.0	81.2	0.013*
		C2	61.8	12.3		60.3	42.7	82.0	
	CON	C1	58.9	6.94	−1.41% ± 4.78	56.9	48.7	75.4	0.505
		C2	58.0	7.35		56.1	47.6	75.4	
					*p* = 0.017*				

Abbreviations: SD – standard deviation; Min – minimum value; Max – maximum value; *p* – probability level for differences between RWL and CON groups; RWL – rapid weight loss group; CON – control group; SQ – squat; BP – bench press; DL – deadlift; IPF GL – IPF Good Lift score; IPF GL BP – IPF Good Lift Bench Press score; TOTAL – sum of the highest successful attempts in SQ, BP, and DL; C1 – first simulated competition; C2 – second simulated competition; * – statistically significant difference (*p* < 0.05); Δ±SD – difference between C1 and C2.

In the RWL group, no participants demonstrated a > 5% increase in squat or TOTAL performance between C1 and C2. One individual showed a > 5% improvement in bench press, and another in deadlift. In the CON group, two participants improved their squat by > 5%, one improved deadlift performance, and one showed a > 5% decrease in deadlift. Only one individual improved their TOTAL by > 5%.

### 3.4. Rapid weight loss and perceived exertion

Subjective ratings of perceived exertion (RPE) across different lifts (SQ, BP, DL) are presented in Table 4. In most cases, no statistically significant differences in RPE were observed between the RWL and CON groups. However, a significant group difference was noted for RPE in the first squat attempt (SQ1) between C1 and C2 (*p* < 0.05).

**Table 4.** Effects of rapid weight loss on perceived exertion (RPE).

Variable	Group	Timepoint	Mean	SD	Δ±SD	Median	Min	Max	*p*
RPE - SQ 1	RWL	C1	7.92	0.96	8.78% ± 13.9	8	6	9	0.056
		C2	8.54	0.88		9	7	10	
	CON	C1	8.46	0.88	−5.27% ± 13.5	8	7	10	0.13
		C2	8.00	1.22		8	5	9	
					*p* = 0.037*				
RPE - SQ 2	RWL	C1	9.08	0.64	−0.24% ± 13.1	9	8	10	1
		C2	9.00	0.91		9	7	10	
	CON	C1	9.31	0.63	3.7% ± 8.14	9	8	10	0.182
		C2	9.62	0.51		10	9	10	
					*p* = 0.589				
RPE - SQ 3	RWL	C1	9.85	0.38	−0.684% ± 5.14	10	9	10	0.773
		C2	9.77	0.44		10	9	10	
	CON	C1	10.0	0.00	−1.54% ± 3.76	10	10	10	0.346
		C2	9.85	0.38		10	9	10	
					*p* = 0.708				
RPE - BP 1	RWL	C1	7.69	1.03	3.44% ± 19.6	8	6	10	0.803
		C2	7.85	1.07		8	6	9	
	CON	C1	7.92	0.95	2.44% ± 17.0	8	7	10	0.76
		C2	8.00	0.82		8	6	9	
					*p* = 0.754				
RPE - BP 2	RWL	C1	9.15	0.69	4.49% ± 7.82	9	8	10	0.089
		C2	9.54	0.66		10	8	10	
	CON	C1	9.23	0.60	0.36% ± 8.89	9	8	10	1
		C2	9.23	0.60		9	8	10	
					*p* = 0.311				
RPE - BP 3	RWL	C1	9.85	0.38	0.86% ± 3.08	10	9	10	1
		C2	9.92	0.28		10	9	10	
	CON	C1	10.0	0.00	0.00% ± 0.00	10	10	10	1
		C2	10.0	0.00		10	10	10	
					*p* = 0.378				
RPE - DL 1	RWL	C1	8.08	1.04	3.05% ± 12.8	8	6	9	0.588
		C2	8.23	0.73		8	7	9	
	CON	C1	8.15	1.28	−5.97% ± 22.0	8	5	10	0.59
		C2	7.77	2.17		9	2	10	
					*p* = 0.528				
RPE - DL 2	RWL	C1	9.38	0.65	3.61% ± 6.96	9	8	10	0.129
		C2	9.69	0.48		10	9	10	
	CON	C1	9.38	1.12	0.68% ± 9.21	10	6	10	1
		C2	9.38	0.96		10	7	10	
					*p* = 0.405				
RPE - DL 3	RWL	C1	10.0	0.00	0.00% ± 0.00	10	10	10	1
		C2	10.0	0.00		10	10	10	
	CON	C1	9.62	0.87	1.95% ± 4.81	10	7	10	0.346
		C2	9.77	0.60		10	8	10	
					*p* = 0.166				

Abbreviations: SD – standard deviation; Min – minimum value; Max – maximum value; *p* – probability level for differences between RWL and CON groups; RWL – rapid weight loss group; CON – control group; SQ – squat; BP – bench press; DL – deadlift; C1 – first simulated competition; C2 – second simulated competition; * – statistically significant difference (*p* < 0.05); Δ±SD – difference between C1 and C2.

### 3.5. Dietary composition during the intervention

Table 5 presents the dietary composition during the intervention phase. At C2, the RWL group had significantly lower total energy intake, lower relative carbohydrate intake, and lower fiber and sodium consumption compared to the CON group. Conversely, fat intake and dietary fat percentage were significantly higher in the RWL group.

**Table 5.** Nutritional composition during the intervention period.

Variable	Group	Mean	SD	Median	Min	Max	p
Energy intake [kcal]	RWL	2640	274	2690	2220	3240	0.003*
	CON	3160	515	3040	2570	4540	
Relative energy intake [kcal/kg]	RWL	28.3	2.65	28.9	22.4	32.8	0.001*
	CON	35.0	5.52	35.3	24.6	46.6	
Protein [g]	RWL	179	22.1	179	140	210	0.878
	CON	176	28.3	180	113	228	
Relative protein intake [g/kg]	RWL	1.92	0.225	1.93	1.53	2.33	0.724
	CON	1.96	0.403	1.97	1.4	2.82	
Fat [g]	RWL	195	24.1	197	162	245	0.001*
	CON	95.3	17.4	100	60.2	123	
Fat percentage in diet [%]	RWL	66.4	2.25	65.4	64.7	70.9	0.001*
	CON	27.3	4.32	28.4	17.9	32.5	
Carbohydrates [g]	RWL	38.3	6.25	36.8	26.9	49.7	0.001*
	CON	362	101	344	237	567	
Relative carbohydrate intake [g/kg]	RWL	0.412	0.0814	0.399	0.305	0.59	0.001*
	CON	4.00	1.05	3.76	2.47	6.13	
Fiber [g]	RWL	8.56	2.68	9.4	0.00	9.9	0.001*
	CON	30.3	13.1	28.6	12.7	55.1	
Sodium [mg]	RWL	1250	75.6	1250	1160	1430	0.001*
	CON	3270	1040	3090	2020	5890	

Abbreviations: *N* – sample size; SD – standard deviation; Min – minimum value; Max – maximum value; *p* – probability level for differences between RWL and CON groups; RWL – rapid weight loss group; CON – control group; * – statistically significant difference (*p* < 0.05).

### 3.6. Rapid weight loss and mood

RWL did not change TA, EA and HT. However, before C2, the RWL was characterized by significantly higher TA values compared to CON (Table 6).

**Table 6.** Effects of rapid weight loss on perceived exertion Tense Arousal (TA), Energetic Arousal (EA), and Hedonic Tone (HT).

Variable	Group	Timepoint	Mean	SD	Δ±SD	Median	Min	Max	*p*
TA	RWL	C1	18.5	4.22	10.4% ± 29.5	18.0	12.0	28.0	0.527
		C2	19.8	4.09		20.0	13.0	27.0	
	CON	C1	17.0	4.93	3.45% ± 24.4	17.0	10.0	30.0	0.623
		C2	16.6	2.53		17.0	12.0	22.0	
					*p* = 0.817				
EA	RWL	C1	27.8	2.73	−3.32% ± 16.8	28.0	23.0	33.0	0.664
		C2	26.8	4.64		27.0	16.0	33.0	
	CON	C1	29.7	3.22	−0.07% ± 15.5	30.0	24.0	35.0	0.894
		C2	29.5	4.84		30.0	23.0	39.0	
					*p* = 0.898				
HT	RWL	C1	30.7	3.68	−2.12% ± 11.0	30.0	24.0	36.0	0.407
		C2	29.8	2.65		30.0	26.0	33.0	
	CON	C1	30.5	6.28	4.09% ± 19.7	32.0	12.0	38.0	0.927
		C2	30.7	3.61		32.0	20.0	33.0	
					*p* = 0.59				

Abbreviations: TA – tense arousal, EA – energetic arousal, HT – hedonic tone, SD – standard deviation; Min – minimum value; Max – maximum value; *p* – probability level for differences between RWL and CON groups; RWL – rapid weight loss group; CON – control group; * – statistically significant difference (*p* < 0.05).

## 4. Discussion

A review of the existing literature suggests that the present study is the first to evaluate whether the combination of rapid weight loss (RWL) strategies – targeting reductions in gut content, muscle glycogen, and body water – followed by a short-term recovery period (~2 hours) affects maximal strength performance in competitive powerlifters.

It is also important to assess whether the observed changes in body composition variables (e.g. body mass, fat mass, fat-free mass) exceeded the measurement error range of the bioelectrical impedance analysis (BIA) device. Based on published minimum difference (MD) thresholds – which correspond to minimum detectable change (MDC) – for the InBody 770 (a device using similar 8-point electrode and multi-frequency BIA technology as the InBody 570), the reductions observed in our study exceeded those cutoffs: body mass (MD = 0.6 kg), fat mass (MD = 1.66 kg), and fat-free mass (MD = 1.59 kg) were all surpassed by the changes recorded (−4.4 kg, −2.7 kg, and −1.8 kg, respectively). Only the reduction in body fat percentage (~2.0 pp) approached but did not clearly exceed the reported MD (MD = 2.24 pp), suggesting caution when interpreting percentage values in isolation [21]. Overall, these results indicate that the observed changes likely reflect true physiological alterations rather than device-related measurement error.

Our findings indicate that such a strategy did not change maximal strength (TOTAL, SQ, BP, DL), aligning with previous research. Brechney et al. [10] demonstrated that although anaerobic capacity, maximal strength, and endurance may decline following a ~ 5% body mass reduction, an appropriate recovery phase can reverse these effects. While we did not assess strength performance immediately after RWL, previous studies show that ~ 3% dehydration can impair 1RM (1-repetition maximum) [7,8], suggesting that the maintained strength in our study may reflect effective recovery.

For example, calorie and fluid restriction led to a ~3 kg decrease in handgrip strength, which returned to baseline after rehydration and refeeding [22,23]. The lack of changes in maximal strength capacity may be explained by the fact that a SQ, BP and DL during competition typically lasts only a few seconds. Therefore, the availability of muscle glycogen is not a limiting factor for performance in powerlifting. During the first three seconds of muscle contraction, phosphocreatine breakdown accounts for approximately 70% of ATP production [24] gradually declining as anaerobic glycolysis becomes dominant after around six seconds of exercise [25]. Additionally, the recovery period planned by a sports dietitian promoted the restoration of euhydration through the consumption of an 800 ml bolus of oral rehydration solution (ORS), followed by regular smaller fluid boluses.

Similarly, Muay Thai athletes were shown to regain strength following recovery, underlining the critical role of this phase. Durguerian et al. [15] also reported that a 4.34% reduction in body mass had no negative impact on TOTAL performance when athletes were allowed to rehydrate and refuel before competition. Wrestlers who lost 5.4% of their body mass also regained performance capacity after 16 hours of recovery [26]. These findings highlight the importance of a well-structured recovery strategy in minimizing the negative effects of dehydration.

Dehydration of 3–4% body mass has been shown to negatively affect strength and endurance [27], though most studies have used testing protocols not specific to powerlifting. For instance, 2–3% dehydration significantly reduced 1RM in bench press, but strength returned to baseline following rehydration [7,8]. Similar observations have been reported in other sports [28], further supporting the benefit of recovery.

In contrast to combat sports, where depleted muscle glycogen stores may impair exercise capacity [29], powerlifting is characterized by very short-duration maximal efforts, with each lift typically lasting less than 6 seconds. During the first three seconds of muscle contraction, phosphocreatine breakdown contributes approximately 70% of ATP production [24], gradually declining as anaerobic glycolysis becomes the dominant energy system after around six seconds of exercise [25]. Therefore, muscle glycogen does not appear to be a limiting factor for maximal strength performance in powerlifting. For example, a 7-day ketogenic diet intervention did not affect handgrip strength, vertical jump height, or 1RM in the squat [6]

In the current study, dehydration was likely the only component of the RWL protocol with the potential to change strength. However, the implemented recovery phase significantly reduced these potential negative changes. Moreover, the use of a nutrition strategy designed by a sports dietitian likely minimized the risk of irrational or unsafe methods [2]. For example, Connor and Egan [30] reported that an MMA athlete who lost 9.1% of body mass without dietitian supervision experienced a decline in handgrip strength after following an extremely low-calorie diet (708 ± 428 kcal/day), 6 L of water intake for 5 days, followed by fasting and fluid restriction. In contrast, an athlete who cooperated with a dietitian and reduced 5.3% of body mass using a more structured plan (1600 kcal/day, 6 days, fasting, and 2 L of water) maintained handgrip strength – and even improved it in one hand.

The results of our study support the importance of recovery strategies – especially appropriate hydration and energy intake – for maintaining maximal strength in powerlifting. A 4.81% reduction in body mass may allow athletes to compete in a lower weight class against opponents with potentially lower TOTAL scores, thereby increasing their chances of winning. In fact, the RWL group showed increased IPF GL and IPF GL BP scores, consistent with previous findings [15]

In powerlifting, increased perceived exertion after RWL may lead athletes to underestimate their load selection, resulting in a lower TOTAL. In our study, the RWL group showed a trend toward higher RPE scores in 7 out of 9 lifts from C1 to C2, although the differences were neither statistically nor practically significant. A meta-analysis by Deshayes et al. [31] showed that 1% dehydration increased RPE by 0.21 units, with significant effects emerging only at > 3% body mass loss. Gann et al. [32] reported a 1-point RPE increase with 3% dehydration in anaerobic exercise, with no effect on performance. Similar patterns were found in resistance training [33] although other studies failed to confirm this relationship [7]. Importantly, RPE scales based on repetitions in reserve (RIR) are considered valid tools for tracking training fatigue [18,34], with a 1-point change often reflecting a meaningful shift in internal load. Therefore, even without changes in absolute strength, elevated RPE in the RWL group may suggest increased physiological strain. However, when euhydration is achieved during recovery, RWL likely has minimal impact on subjective exertion during strength performance.

RWL did not significantly change mood dimensions such as Tense Arousal (TA), Energetic Arousal (EA), or Hedonic Tone (HT); however, an increase in TA was observed in the experimental group (RWL) before the second simulated competition. Interestingly, similar findings were observed in endurance athletes. In a study by [35], participants reported elevated TA levels before a nighttime ultramarathon, which may reflect a state of stress, nervousness, or tension in anticipation of competition. This increase in TA could therefore be interpreted not necessarily as a negative psychological response, but rather as a form of competitive arousal. On the other hand, athletes often associate rapid weight loss with their athletic identity. In interviews with 14 members of the Swedish national combat sports team, the RWL process was described as part of their identity, reflecting personal responsibility and contributing to a sense of mental advantage [36] Although greater TA in the RWL group could theoretically predispose athletes to lower strength outcomes, the current study showed that elevated TA did not impair maximal strength performance in powerlifters. This suggests that when guided by a dietitian, RWL may not negatively impact mood or psychological readiness in powerlifters. These findings contrast with previous studies [15,37], where athletes managed the weight-cutting process independently, possibly leading to greater psychological strain due to less structured and potentially more extreme strategies.

For example, in a study by Nolan et al. [2] 43.5% of powerlifters reported never using a low-residue (low-fiber) diet. Additionally, 19% of athletes “always” or “sometimes” used saunas, 31% relied on hot/salt baths, and 13% reported the use of diuretics. Each of these strategies carries a higher risk of impaired mood and performance compared to the less invasive approach of low-fiber dieting. Therefore, dietitian supervision facilitates the selection of the most effective and least detrimental RWL strategies, helping to preserve both physical capacity and psychological state.

While our study focused on relative (IPF GL) and absolute (TOTAL) performance changes following rapid weight loss, recent normative datasets – such as those presented by van den Hoek et al. [38] provide valuable insight into general trends in maximal strength levels across weight classes and age categories in competitive powerlifters. Although these reference values are not directly comparable with our intervention-based findings, they contribute to a broader understanding of performance standards in the sport.

Overall, this study supports the feasibility and safety of short-term rapid weight loss (RWL) strategies, provided they are paired with a well-designed and properly executed recovery protocol. Such an approach enables athletes to qualify for a lower weight class while maintaining maximal strength performance. Consequently, this may yield a relative competitive advantage – such as higher IPF GL scores – thereby increasing the likelihood of success in both weight-class-based rankings and open categories.

Future research perspective

Although the current findings provide valuable insights into the effects of RWL in men under short-term recovery conditions, future studies should also include female participants, as their physiological and hormonal responses to body mass reduction protocols may differ. Moreover, given the longer time interval between weigh-in and competition in other powerlifting federations, it would be worthwhile to compare the effects of different magnitudes of RWL (e.g. 5–10%) combined with varying recovery durations (e.g. 2–24 hours).

### 4.1. Study limitation

This study has several limitations that should be acknowledged. Although maximal strength was assessed through a simulated competition setting, the lack of direct 1RM testing under controlled laboratory conditions may not reflect actual maximal strength capabilities. Additionally, the RWL protocol and dietary implementation were carried out in home settings without direct supervision from dietitians, which could have introduced variation in adherence and execution of the intervention. A key limitation of this study is the lack of post-recovery body weight measurements. As body mass was only assessed prior to the RWL protocol and at weigh-in, we were unable to quantify the extent of weight regained during the 2-hour recovery period. This information would have provided greater insight into the actual competition weight and the effectiveness of the rehydration and refeeding strategy. The short recovery period (~2 hours) reflects standard IPF weigh-in conditions but may limit extrapolation to federations with longer recovery windows. In federations with extended intervals between weigh-in and competition, athletes may have the opportunity to lose a greater amount of body mass (5–10%) without compromising maximal strength performance. This could potentially allow them to compete in even lower weight classes and further enhance their relative strength scores. Furthermore, while the sample size was statistically adequate, it remains relatively small, which may affect the generalizability of the results, particularly to other competitive levels. Lastly, this study included only male participants, and the findings may not be directly applicable to female powerlifters, who may respond differently to RWL protocols.

Although effect size estimates (Cliff’s delta and rank-biserial correlations) were included to enhance the interpretability of between- and within-group differences, their precision may be limited due to the small sample size, and they should be interpreted with caution.

## 5. Conclusion

In the group that underwent a rapid weight loss of approximately 5%, followed by a short-term recovery period, maximal strength performance was maintained. This indicates that it is possible to reduce body mass without compromising absolute strength levels in competitive powerlifters. Moreover, the increase in IPF GL and IPF GL BP scores observed after the intervention suggests an improvement in relative scoring metrics, while maximal strength was maintained. This may offer a competitive advantage in open-category competitions. These findings support the potential use of well-planned weight reduction protocols – implemented under professional guidance and combined with structured recovery strategies such as rehydration and energy replenishment – as a viable strategy for improving relative performance and competitive positioning in strength sports.

## Supplementary Material

Supplemental Material

## Data Availability

The data underlying this article will be shared on reasonable request to the corresponding author.
