# Supplementary material for: Making weight makes sense: relative performance gains after rapid weight loss in powerlifting: a randomized controlled trial
Source: J Int Soc Sports Nutr. 2025 Aug 27;22(1):2550309. doi: 10.1080/15502783.2025.2550309 (PMC12392435; doi:10.1080/15502783.2025.2550309)
Supplement: Supplemental Material [file RSSN_A_2550309_SM5849.docx]

**Supplementary material**

Table S1. Cliff’s delta values for baseline characteristics between RWL and CON groups

| **Variable** | **Cliff’s delta** |
| --- | --- |
| Age [years] | 0.343 |
| Height [m] | −0.089 |
| Body mass [kg] | 0.077 |
| Maximal training performance (TOTAL) [kg] | 0.249 |
| Body fat percentage [%] | 0.231 |
| Training experience [years] | 0.089 |
| Number of powerlifting competitions participated | 0.130 |

**Abbreviations**: RWL – rapid weight loss group; CON – control group;

TOTAL – sum of the heaviest successful attempt in squat, bench press, and

deadlift (training data);

Table S2. Effect size estimates (*r_c_* and Cliff’s delta) for body composition changes within and between groups

| **Parameter** | ***r_c_* RWL** | ***r_c_* CON** | **Cliff’s delta** |
| --- | --- | --- | --- |
| Body mass [kg] | −1.000 | −0.103 | −0.976 |
| Total body water [%] | −0.934 | 0.551 | −0.822 |
| Fat mass [kg] | −1.000 | −1.000 | −0.817 |
| FFM [kg] | −1.000 | 0.576 | −0.888 |
| Body fat percentage [%] | −1.000 | −1.000 | −0.769 |

**Abbreviations**: RWL – rapid weight loss group; CON – control group; FFM – fat-free mass;

*r_c_* – matched-pairs rank biserial correlation coefficient (within-group effect size);

Table S3. Effect size estimates for maximal strength performance (SQ, BP, DL, TOTAL, IPF GL)

| **Parameter** | ***r_c_* RWL** | ***r_c_* CON** | **Cliff’s delta** |
| --- | --- | --- | --- |
| SQ [kg] | −0.667 | 0.327 | −0.308 |
| BP [kg] | 0.036 | 0.289 | −0.130 |
| DL [kg] | −0.091 | 0.028 | −0.065 |
| TOTAL [kg] | −0.227 | 0.422 | −0.367 |
| IPF GL score | 0.846 | −0.026 | 0.568 |
| IPF GL BP score | 0.821 | −0.231 | 0.556 |

**Abbreviations**: RWL – rapid weight loss group; CON – control group; SQ – squat; BP – bench press;

DL – deadlift; TOTAL – sum of the heaviest successful attempt in squat, bench press, and deadlift;

IPF GL – International Powerlifting Federation Goodlift Score; IPF GL BP – IPF GL for bench press;

*r_c_* – matched-pairs rank biserial correlation coefficient (within-group effect size);

**Table S4.** Effect size estimates for Rate of Perceived Exertion (RPE) scores

| **Parameter** | ***r_c_* RWL** | ***r_c_* CON** | **Cliff’s delta** |
| --- | --- | --- | --- |
| RPE – Squat 1 | 0.821 | −0.714 | 0.467 |
| RPE – Squat 2 | −0.028 | 0.500 | −0.130 |
| RPE – Squat 3 | −0.333 | −1.000 | 0.065 |
| RPE – Bench press 1 | 0.111 | 0.133 | −0.047 |
| RPE – Bench press 2 | 1.000 | 0.000 | 0.237 |
| RPE – Bench press 3 | 1.000 | 0.000 | −0.006 |
| RPE – Deadlift 1 | 0.250 | −0.286 | 0.112 |
| RPE – Deadlift 2 | 0.667 | 0.000 | 0.195 |
| RPE – Deadlift 3 | 0.000 | 1.000 | −0.154 |

**Abbreviations**: RWL – rapid weight loss group; CON – control group; RPE – Rate of Perceived

Exertion; *r_c_* – matched-pairs rank biserial correlation coefficient (within-group effect size);

# Table S5. Cliff’s delta values for nutritional intake variables during intervention.

| **Parameter** | **Cliff’s delta** |
| --- | --- |
| Energy intake [kcal] – measurement 1 | −0.065 |
| Relative energy intake [kcal/kg] – measurement 1 | −0.065 |
| Protein [g] – measurement 1 | −0.278 |
| Relative protein intake [g/kg] – measurement 1 | −0.314 |
| Fat [g] – measurement 1 | 0.195 |
| Dietary fat percentage [%] – measurement 1 | 0.302 |
| Carbohydrates [g] – measurement 1 | −0.183 |
| Relative carbohydrate intake [g/kg] – measurement 1 | −0.231 |
| Fiber [g] – measurement 1 | −0.142 |
| Sodium [mg] – measurement 1 | −0.266 |

**Abbreviations**: RWL – rapid weight loss group; CON – control group;
